# Accelerating Innovation in the Creation of Biovalue: The Cell and Gene Therapy Catapult

**DOI:** 10.1177/0162243917702720

**Published:** 2017-04-06

**Authors:** John Gardner, Andrew Webster

**Affiliations:** 1 School of Social Sciences, Monash University, Clayton, Melbourne, Victoria, Australia; 2 Science and Technology Studies Unit, Department of Sociology, University of York, Heslington, York, United Kingdom

**Keywords:** futures, alternative life forms, markets/economies, politics, power, governance

## Abstract

The field of regenerative medicine (RM) has considerable therapeutic promise that is proving difficult to realize. As a result, governments have supported the establishment of intermediary agencies to “accelerate” innovation. This article examines in detail one such agency, the United Kingdom’s Cell and Gene Therapy Catapult (CGTC). We describe CGTC’s role as an accelerator agency and its value narrative, which combines both “health and wealth.” Drawing on the notion of sociotechnical imaginaries, we unpack the tensions within this narrative and its instantiation as the CGTC cell therapy infrastructure is built and engages with other agencies, some of which have different priorities and roles to play within the RM field.

## Introduction

Regenerative medicine (RM) has been defined as that which “replaces or regenerates human cells, tissue and organs, to restore or establish normal function” (Mason and Dunnill 2008, 4). This is considered to be revolutionary when compared to conventional treatments based on drugs or devices, and it is widely claimed that RM will have the potential to provide curative treatments for a range of illnesses, such as diabetes, heart disease, and various neurological disorders (Department for Business, Innovation & Skills 2011). There is, then, considerable high expectation about RM’s clinical potential (Morrison 2012).

Clinical promise surrounding RM is accompanied by highly optimistic claims about its economic impact. RM, it is claimed, will become the basis for a thriving industry that will underpin a high-wealth, knowledge-based economy. The Japanese government, for example, has named RM as a pillar of its economic growth strategy (Ogawa 2015), and the promise of economic growth was a motivation for the State of California’s “Proposition 71: The Californian Stem Cell Research and Cures Initiative” (Longaker, Baker, and Greely 2007). In the UK, RM was named by the British government as one of the “eight great technologies” that will drive economic growth within the UK and which has the potential to become a multibillion pound industry (Willetts 2013). As with each of the “great” technologies, RM has been identified as an area in which the UK can excel, but in which targeted public investment will be needed to convert a strong science base into a wealth-generating industry. This is part of a broader discourse on “health and wealth” in the UK involving close alignment of biomedical researchers, the health-care system, and the commercial sector (Shaw and Greenhalgh 2008).

At the same time, however, there are concerns that the potential of RM to deliver curative treatments and generate wealth will be hindered by an array of innovation challenges (Gardner et al. 2015; Gardner and Webster 2016). Currently, there are few RM therapies available to patients, which some commentators argue reflects an incommensurability between the health-care system and the exigencies of the nascent RM field (Tait 2007; Omidvar et al. 2014). Specific concerns relate to the instability of live tissues and cells, manufacturing scale-up and logistical difficulties, the burden of current regulatory arrangements, securing reimbursement (payment), and the challenge of integrating novel procedures into existing day-to-day health-care workflows. In several countries, this discourse of concern has prompted the formation of publicly funded, innovation “accelerator” agencies whose aim is to promote both a faster route to the clinic and a more rapid valorization of product. These include, for example, the New York State Stem Cell Science, the Canadian Centre for Commercialization of Regenerative Medicine, and, most significantly, the California Institute for Regenerative Medicine, established in 2004 with a budget of USD3 billion for RM research, infrastructure, and training.

In the UK, various initiatives have been taken that are designed to overcome the perceived innovation challenges in RM (Department for Business, Innovation & Skills 2011; Regenerative Medicine Expert Group 2015; House of Lords Science and Technology Committee 2013; UK Research Councils 2012). The most significant initiative has been the innovation accelerator agency, the Cell and Gene Therapy Catapult (CGTC). The CGTC was established in 2012, and it has been allocated £70 million in public funds to help support the development of an RM industry in the UK. Such initiatives raise important questions for the field of science and technology studies: how do intermediary agencies such as the CGTC “accelerate” innovation? How do they attempt to build and mobilize markets and the infrastructure that can support them? And, more importantly, what tensions might be there in the narrative of health and wealth, which drives these processes in the UK (and indeed elsewhere)? In this article, we address these questions by undertaking the first detailed examination of the role of the CGTC in the field of RM.

Using data from both fieldwork and secondary sources, and by drawing on Jasanoff and Kim’s (2009) concept of “sociotechnical imaginaries,” we examine the value assumptions embedded in specific visions and expectations of the RM future and how the CGTC is implicated in this process. We are especially interested in those practices that are designed to accelerate innovation and facilitate the emergence of a wealth-generating RM industry. As part of this, the CGTC has a key social role in *legitimating* particular RM projects and products that it regards as being commercially promising. We show how the enactment of the sociotechnical imaginary of the CGTC depends on the play of––and tensions between––distinct sociotechnical networks (Callon 1999), which then have important implications for the realization of commercial biovalue (Waldby 2002), such that some products may “accelerate” toward the market more readily than others. Thereby, the realization of the twin virtues of health and wealth is not so easily achieved: competing values and priorities complicate the innovation journey.

## Intermediary Agencies and the (Accelerated) Generation of Value

Intermediary agencies in the RM field create diverse forms of value that serve different purposes and users. A contrast can be made, for example, between the CGTC and another influential institution in the RM landscape, the UK Stem Cell Bank (UKSCB). The UKSCB, which has been extensively explored (Stephens, Atkinson, and Glasner 2008, 2013; Stephens, Lewis, and Atkinson 2013), was established in 2004, and its creation was in response to a widespread concern at that time about the ethical procurement of human embryonic stem cells (hESCs). The bank was tasked with mitigating such concerns by establishing an ethical repository of hESC lines for research and clinical purposes. It seeks to do this through managing and facilitating the ethical sourcing and use of cell lines as either research (i.e., for experimental lab-based work) or clinical (i.e., for therapeutic intervention) grade lines. Like the UKSCB, the formation of the CGTC was prompted by prevalent political concerns of the time, which were more to do with markets than with ethics. The CGTC was formed in the hope that it would facilitate the flow of tissues and cells, expertise, and investment that would be required to ensure that the UK’s “excellent basic science base” would be translated into useful, commercially successful therapies (Thompson and Foster 2013). Unlike the UKSCB, however, which deals in lines whose comparability (and therefore value) derives from their being *standardized* and *ethically procured*, the CGTC seeks to create and define the value of cell lines/therapies by calculating and subsequently creating *their marketability*. This requires the construction of future-oriented visions that are allied to considerable organizational labor and resources through which a manufacturing platform and related services can be put in place.

Jasanoff and Kim’s (2009) notion of “sociotechnical imaginaries” provides a useful framework for understanding the role of intermediary innovation agencies such as the CGTC and the UKSCB. Sociotechnical imaginaries are collectively produced visions of social life that are reflected in the design of current sociotechnical projects. These visions may be future-oriented, and they encode particular understandings of what constitutes a “good society” (Jasanoff and Kim 2009, 123). By being enacted in the present, they also have productive, structuring effects in the present: as with promissory expectations more generally (Borup et al. 2006), institutional imaginaries delineate social roles and coordinate alliance-building activities. Stephens, Atkinson & Glasner (2013), for example, argue that the UKSCB enacts a particular *institutional sociotechnical imaginary*. Specifically, its institutional structure and governance model is designed to reassure UK publics that its activities are ethical and serve a public good role. This requires that it be seen as a trustworthy institution, and it entails a set of activities that validate certain researchers and certain laboratories and clinics as being ethical producers or users of hESC (and other) lines stored in the bank. As a result, networks of tissue flow become validated as “ethical.” It is through such work that a socially legitimated future for stem cell medicine is proposed (Stephens, Atkinson, and Glasner 2008).

In a similar vein, the CGTC reflects particular sociotechnical imaginaries, though they are aligned not with ethics but with notions of health and wealth, ostensibly twinned national interests combining values associated with the public (population health) good *and* the private market. This, in turn, requires the formation of a durable infrastructure through which such an imaginary can be framed and mobilized (Callon 1999). The CGTC infrastructure is based on the sociotechnical imaginary of the “catapult” model that was originally created in 2010 by the Technology Strategy Board (now known as Innovate UK), a public agency which reports to the Department for Business, Energy, and Industrial Strategy. There are currently nine catapults that have been formed to bring together expertise and resources in diverse science and technology areas (such as transport, digital technologies, energy systems, etc.), all of which have been identified as having the potential to drive––or to catapult––economic growth. They are described as being nonprofit, business-led centers that connect business with the UK’s research and academic communities.

The CGTC, established 2012, is based in London and has approximately a hundred staff. According to its webpage, its stated aim is to

Lead the UK cell therapy industry to create health and wealth from the UK’s outstanding science foundation and make the UK the most compelling and logical choice for our international partners. (CTC 2015a)

This emphasis on creating health and wealth in building a new RM industry is reflected in the personnel who comprise senior staff in the CGTC. The board of directors has six members from the biotech, pharma, and life sciences industry and two members with academic science backgrounds. All five members of the management team have a professional background in the life-sciences industry, and the advisory group includes eight representatives from various companies (including AstraZeneca, GlaxoSmithKline, GE HealthCare, and Johnson & Johnson) and seven stem cell scientists.

In general terms, the CGTC positions itself as an innovation accelerator, as expressed in four linked activities that reflect a linear path from upstream to more applied innovation. First, it aims to facilitate “upstream” innovation by helping universities to “capture [the] value” of their academic research in the field. CGTC staff say they have “crisscrossed” the UK, screening universities for promising RM projects, with the intention of facilitating linkages between academic researchers, university technology transfer officers, and industry. This has involved identifying the key challenges to innovation at this upstream stage, one of which is focused on identifying and managing intellectual property (IP). Hence, the CGTC established an IP and access scheme “to capture the value of [universities’] novel IP” (Herbert 2014, 15). The CGTC also collaborates with academic researchers on grant applications (in part to help secure additional funding for itself), and it actively supports RM conferences (CTC 2014b). Second, the CGTC seeks to support clinical development, clinical trialing, and navigating regulation. They collaborate with academic and commercial partners to push projects into the clinical trial phase of development by providing advice on navigating the trials process and, in some cases, by acting as a clinical trials sponsor (ensuring that a trial is appropriately funded and managed). Such support is thought to be especially important for those small companies that dominate the RM landscape and lack the necessary in-house skills and resources to do this independently. Third, and closer to more applied processes, the CGTC supports RM manufacturing and logistics operations, which include clean-room/laboratory space for rental by users and in which cell and tissue manufacturing processes can be trialed according to legally defined quality standards. Finally, the CGTC’s expertise in finance, health-care economics, and business models is intended to assist partners in formulating an RM product development pathway that takes into account assumptions about target markets and anticipated innovation challenges in order to build RM businesses “with investable propositions” (CTC 2014b, 7). More broadly, the CGTC plays something of a lobbying role for those working within the field of RM, pushing for the regulatory and policy adjustments that are believed to facilitate innovation in the field. Overall, then, as an accelerator agency, the CGTC illustrates what Salter and Faulkner (2011, 1-2) refer to as the established policy orthodoxy in “competition” states such as the UK; an orthodoxy in which the state––through agencies like the Catapult––aims to “foster the conditions necessary for innovation,” “stimulating a dynamic” that enables a field eventually to become self-sustaining, rather than (as in the era of corporatist industrial strategy) directly sponsoring particular firms or technology sectors.

In these ways, the CGTC acts to steer what might otherwise be dispersed, heterogeneous, and uncoordinated research and clinical activity into particular paths of RM commercialization, aimed at generating market value. The CGTC can be seen as the materialization and institutionalization of a set of promissory expectations (Borup et al. 2006) about the clinical and economic value of biological material. These expectations are combined with broader assumptions about the role of the private and public sectors in innovation and wealth creation, a neoliberal agenda that assigns to knowledge and health a market value and sees the “public good” as coinciding with market commercialization. Together, these expectations and assumptions form a sociotechnical imaginary grounded in the notion of accelerated innovation. As we shall see, however, biological material––the cell line, for example––is immersed in various “entanglements” that encompass a range of other actors. Such entanglements are indicative of the tensions that arise within the “competition state” (Salter 2009), in which a diversity of actors, expertise, and knowledge are mobilized in an attempt to generate health and wealth. We explore these in the fourth section.

## Method

We draw upon data that have been collected as part of a larger social science project funded by the Economic and Social Research Council, exploring the social dynamics of innovation within the field of RM. The project involved over eighty interviews with stem cell scientists, clinicians developing RM therapies, regulators, patient association representatives, industry representatives, health economists, and public servants working within the health-care system. Elsewhere, we have published work relating to the specific factors shaping the adoption of RM in distinct clinical contexts (Gardner and Webster 2016; Gardner et al. 2017). In this article, we draw specifically on a subset of this much larger data set where respondents reflect upon their engagement with the CGTC. Several of these respondents are professionally associated with the CGTC, while others have engaged with the CGTC in seeking advice or assistance. This article has also been informed by our field notes of several RM-industry conferences and workshops that we attended, and it draws upon various forms of secondary data. These include CGTC webpages, annual reports and other official publications, and the publicly available reports and minutes of other agencies in which the CGTC is mentioned and that provide useful background on the CGTC itself. Ethics approval for the data collection activities was obtained from the relevant institutional ethics committees, and informed consent was obtained from all participants. In the next section, we discuss two of the central processes associated with the accelerated generation of products that are shaped by the health and wealth social imaginary: the selection and positioning or configuring of potential products and the endeavors directed at purifying and scaling them up for the market. We then examine how both processes come into tension with other players and values at work in the field.

## Findings

### Configuring Products for Health and Wealth

A key role played by the CGTC is its direct engagement with firms (mostly but not exclusively UK-based) to help identify those products that are more likely to be effectively configured and positioned for an RM market. These are products that are often at an early stage of development. In terms of the trials process, they are typically at the preclinical phase, or phase I or II, and so they are at a point when a potential product moves toward early efficacy tests and, depending on the results of such tests, manufacturing scale-up. In some cases, the Catapult will enter into collaborative “core” partnerships with smaller companies and organizations with “medium-” or “high-risk” projects (CTC 2015a), which may last several years. Currently, the CGTC is involved in over forty projects with companies or organizations, and it is currently sponsoring two RM clinical trials. In this section, we focus on those activities aimed at early stage product development. We draw on secondary data and interviews with individuals who have been involved in projects receiving CGTC assistance, and we show how these activities attempt to position and configure a prospective RM product for an envisaged RM market.

The business development and market access activities of the CGTC bring together and focus its broad expertise on a specific company or organization with a novel and potentially promising technology. The organization and the technology are subject to detailed scrutiny and assessment of its perceived commercial value. Below, a scientist working in immunotherapy describes this process:

So the [RM work] that we’ve been doing…[the CGTC] actually invested a lot of time and effort in preparing a report on the strengths and weaknesses of the technology, of how to drive it forward, of what the gap analysis was. Our intellectual property and its strengths and weaknesses there. The market that there would be. So they actually put a lot of effort into producing a very detailed report on our technology, which has been very helpful…the purpose of the report that they prepared for us was to identify what the needs were in translating it to the clinic. So because of that report we have a much better idea of the way forward and we’ve been trying to raise the money to actually take it forward ever since. (Scientist 1)

Several of our respondents reported similar engagement with the CGTC, and as the quote illustrates, this engagement entails foregrounding and assessing particular parameters through which value is to be configured. These relate to determining what is described as the “value proposition” of the technology, and hence the associated potential market of the technology, the possible pathway for further developing the technology, and an assessment of its immediate and longer-term IP value. These parameters, of course, reflect the assumptions and professional background of the CGTC staff, and in foregrounding these (and so discounting others), the CGTC acts to produce a vision of the future centered on commercialization and wealth creation. In effect, this sociotechnical imaginary becomes––via its institutionalization in the CGTC––a structuring principle for the production of future-oriented visions for specific RM projects, thus steering mechanisms that overtime are likely to foster a product’s path dependency as it moves toward the market. A core feature of these future-oriented visions is the delineation of the anticipated *use* of the technology, that is, how it will address a particular clinical or research need in an anticipated future. In this way, we see the narrative of “RM as health-generating industry” coming into prominence. Companies and organizations will have some idea of the prospective clinical use value and the “pathway to the clinic” of their technology prior to their engagement with the CGTC, but the activities of the CGTC can be seen as adjusting, further delineating, and reifying particular visions of that future.

As the sociology of expectations literature has illustrated, promissory visions of the future have performative effects 2000 (Brown, Rappert, and Webster 2000; Borup et al. 2006). This is apparent in the way in which a CGTC-mandated prospective clinical use value adds reputational authority to the emerging product or therapy. As the respondents below note, for example, the involvement of the CGTC provided their projects with credibility, which in turn garnered financial assets that could be deployed early in their work:

So the Cell [and Gene] Therapy Catapult were important to us…they gave [our company] and the management team I guess a vote of confidence by putting their support behind us…now we have the backing of the Cell [and Gene] Therapy Catapult who were an organization set up essentially by the TSB and the government to promote and accelerate regenerative medicine in the UK and support companies like us. So that was a huge boost for us and a tick in the credibility box. It enabled us to get the [agency] grant at that time. (CEO—small company)

They have also supported various grant applications that we’ve submitted with letters of support saying how they, you know, think this is a ground breaking technology and so on. (Scientist 1)

As the extracts show, this credibility can be leveraged to secure additional funding, enabling the project to progress further along the envisaged translational pipeline. Importantly, as the CEO’s comment above indicates, the CGTC’s social role as “validator” depends on its reputation. Due to its association with the Technology Strategy Board (TSB), and due to the perceived expertise of its personnel, the CGTC appears to have been, at least among some stakeholders, endowed with the authority to judge the commercial viability of RM projects. Hence, the CGTC adds authoritative weight to prospective clinical values and legitimates particular visions of the future, which thus have a stronger performative impact in the present.

### Purifying Products: Disentanglement and Framing

Commodities and the markets within which they are exchanged have to be actively created and maintained by different agents. They require sociotechnical networks that facilitate types of flows of goods and services. These networks, Callon (1999) has suggested, play a key role in *disentangling* and *framing* entities so that the latter are enacted as commodities; network agents seek to obscure, elide, or sever material and semiotic associations (disentanglement) that could hinder their extraction and exchange; and they establish and foreground (frame) other material and semiotic associations that delineate their relative value, enable their mobility, and thereby promote their consumption. As we have seen in the preceding section, the CGTC is one agent within an emerging RM network that aims to do this. This is apparent, for example, in its role in establishing the IP for a novel therapy or product: IP claims (e.g., through patent filing) serve to set out the specific knowledge claims and supporting methods on which they depend, discrete, or disentangled from the current “state of the art” that thereby demonstrates their novelty.

The delineation and validation of the possible economic and clinical value of a product can be seen as part of this disentangling and framing process. However, there are other entanglements with various degrees of obduracy that the CGTC has to consider. A major role of the CGTC is to facilitate the production of the infrastructure that will enable the industrial scale production of cell and tissue-based products (CTC 2015b). A challenge in this regard for any developer of RM products is the material–physical entanglement of biological material. Cells and tissues are immensely sensitive to their surrounding niche, small changes that can drastically affect their clinical quality, potency, and safety. Cell- and tissue-based RM technologies must, according to the US Food and Drug Administration (FDA) and the European Medicines Agency (EMA) regulatory frameworks, be manufactured in clean-room facilities that meet strict Good Manufacturing Practice (GMP) standards. Cells and tissues need to be carefully procured, expanded, and assayed to ensure they meet quality standards and then carefully distributed to the point of care. These processes generally involve the physical disentanglement of cells and tissues from their original locus (bone marrow aspirate, adipose tissue, etc.) to secure their mobility, the maintenance of some degree of cell-niche physical entanglement necessary to keep cells viable, the introduction of new physical entanglements (such as growth factors, adhesive surfaces) to encourage expansion and differentiation of cells or the selection of desired cell types, and the shielding from unwanted entanglements (contaminants). Currently, these processes often involve labor-intensive “open systems” whose activities are conducted by staff with the appropriate expertise. In order to produce the larger quantity of cells that would be needed for phase III trials or commercial use, less-labor intensive, “closed systems” need to be developed (using bespoke bioreactors, for example), in which some processing steps are automated. Some automated devices have been developed for forms of cell processing, but the use of these needs to be validated by regulators (see Addison 2017). Most importantly, developers must ensure the stability and purity of the product such that it does not carry any “adventitious agents” (i.e., potential biological material such as prions, bacteria, viruses, etc.) that would bring harm to a patient. The costs—especially for developing closed processing systems that can achieve such levels of purification and stability of cells and for maintaining a GMP facility––are high and are said to represent a major translational challenge in RM. Additionally, the existing GMP facilities within which these disentanglement/entanglement processes take place are small and cater to the production of small-scale production of RM technologies needed in phases I and II clinical trials (which involve small numbers of participants). They lack the capacity, then, to produce the vast quantities of cells and tissues that would be needed for larger phase III trials or for commercial use (HoL 2013).

These sociotechnical demands––found across the RM field––pose considerable challenges to the CGTC’s role as an accelerator agency and its drive toward health and wealth. The Catapult has sought to build internal expertise in technology transfer, scale-up, supply chain management, logistics, and GMP. In its collaborations with commercial/academic organizations, the Catapult aims to develop and improve production and logistics processes, and it provides access to their existing manufacturing space to test new systems (CTC 2015b). As a demonstration of the power of the sociotechnical imaginary embodied in the CTGC, it has begun construction of a 5,000 m^2^ publicly funded manufacturing facility (at a cost of £55 million). The CGTC believes that the facility will provide adequate capacity for large-scale production of cell and gene therapies (CTC 2014a) as the need for such facilities grows in the envisaged future.

The facility can be seen as an attempt to create the infrastructure that will support the large-scale disentanglement/entanglement that is necessary for the commercialized flow of live tissues from places of production to places of consumption. It is a material manifestation of promissory expectations and reflects particular understandings about relationships and activities that are needed to enable translation of RM technologies to the clinic. As the CGTC user requirements brief states (CTC 2014c), the facility is being designed so that it enables:

a) current UK clinical manufacturing programs to access the space as demand for scale increases with clinical and commercial maturity; b) product developer companies to produce their own products to help ensure maximum value is retained within the company; c) global companies with more clinically advanced products to locate in the UK to supply EU and, where possible, global markets. (p. 5)

The facility is currently under construction in Stevenage with easy access to London Heathrow airport, the only airport in the UK that, according to the CGTC, has sufficient connectivity to enable the quick transport of RM products to clinics throughout the world (CTC 2014c). Its proximity to London will also ensure access to highly trained staff, and it is being constructed within a life sciences park that has existing infrastructure to ensure ample power supply, waste disposal, and other amenities. The facility will be GMP licensed, and it will be composed of self-contained modules (clean rooms) that can be hired by companies that would otherwise lack the resources to produce their own large-scale facilities. The modules are being designed as spaces that are both rigid and flexible: rigid in certain aspects to maintain strict GMP standards (such as carefully controlled airflow, and segregated flows of wastes, input materials, and so on) and highly flexible in other aspects, so that individual companies can adjust the space as needed depending on the required production processes that, it is envisaged, will change and improve over time. The facility is also being designed to ensure several companies can use the facility at once while protecting commercially sensitive information and practices: personnel access and movement around the facility is to be carefully controlled, and the physical partitioning between modules will enable entanglement/disentanglement processes and know-how to be kept confidential. The first phase of the construction process, which includes the construction of six modules, is expected to be completed in 2017. The facility represents an attempt to establish the conditions for the large-scale, commercial derivation of biovalue––the “yield of vitality produced by the biotechnical reformulation of living processes” (Waldby 2002)––that can thus be directed toward the realization of health and wealth.

Beyond the domain of the CGTC itself, at a wider international level, several RM products have moved sufficiently along the translational pathway to obtain regulatory authorization. Indeed, within the EU, eight RM products have been deemed safe and effective via the Advanced Therapy Medicine Product framework and can now be marketed by their manufacturers. Yet, none of these products has been integrated in health-care systems in a widespread way: the biovalue of RM products (pertaining to both clinical and commercial value) remains largely “promissory” in nature. In South Korea, for example, sixteen RM therapies have received regulatory approval, yet none of these is exported or reimbursed outside of the country (Faulkner 2016). In the following section, we explore why this is the case by drawing attention to some of the other present-day entanglements that complicate the activities of the CGTC and translation activities in RM more generally, and which make the process of acceleration––and the realization of a sociotechnical imaginary––much more problematic.

### Entanglements and Values in Tension

As noted above, the innovation-accelerating activities of agencies such as the CGTC are complicated by other actors that populate the RM field. In effect, these actors are implicated in entangling biological material in various ways, some of which enact values or visions of the future that are potentially in tension with the consolidation of clinical and market value. These derive from and are mobilized by different networks that cut across the CGTC’s activities. We describe below some of the problematizing––and sometimes countervailing––values that are at work and then give some examples of the impact that this can have on prospective products. Our subsequent and concluding discussion will reflect on which of these processes is most likely to pose problems for the realization of the CGTC’s sociotechnical imaginary.

Some forms of entanglement relate to the biological material used in upstream innovation activities, such as the acquisition of, and investment in, cell lines. For example, tissues derived from human embryonic material are entangled in morally charged understandings that have shaped the EU legal landscape for RM. One important issue relates to those legal instruments that permit private ownership and exclusive rights to particular types of “inventions” (thus enabling maximum market value to be retained within a company). These various legal instruments are themselves a reflection of expectations about how innovation can and should be done and where rewards may accrue (Sandor and Varju 2013). Within the EU, a key legal instrument in the field of RM is the so-called Biopatent Directive,^1^ which permits private ownership of particular types of inventions derived from biological entities, but not inventions derived from hESC. This latter exclusion reflects the perceptions of a specific set of actors within the EU (particularly within the European Parliament) that the embryo is a morally privileged entity due to its potential to become a “human.” The directive, then, reflects and enacts a form of value, which could be called “moral value,” and which is in tension with commercialization. It has been the subject of some criticism (Gilbert and Lees 2012), as commentators have felt that it would discourage commercial investment within the EU and companies would direct their attention to jurisdictions such as the United States, where hESC-derived technologies can be patented. While some companies have persisted in developing hESC-derived technologies within the EU, others have deliberately avoided the hESC route to the market. This was the initial strategy of UK-based ReNeuron, which used fetal brain tissue to develop its ReN001 stem cell therapy for the treatment of strokes, patenting the expansion and processing technologies used to produce them.

Another related entanglement that may complicate the commercialization of biological material relates to the debate concerning public good versus private ownership. Some publicly funded tissue banks in the RM field, such as the UKSCB, have procured tissue deposits on the understanding that they would be public goods––that is, that they would be made available as an international resource to any researchers who meet specific ethical criteria. In effect, such banking practices enact tissues as having a public good value due to their capacity to generate knowledge, and so priority is given to research that will use, test, and thus further characterize banked cell lines. Researchers and companies who obtain cells from such banks cannot claim exclusive use for them, and, more significantly, those who deposit lines in the bank must make them available to third parties. The bank grants nonexclusive, royalty-free research licenses for cell lines, without the right to sublicense. Commentators have suggested that the commercial sector will be reluctant to invest in cell-based research and technologies because they cannot guarantee exclusive access to the lines they have deposited. As an interviewee stated:

They’d invest in the acquisition of stem cell lines, only for others to benefit from those lines. (Interview, IP consultant)

Other forms of entanglement relate to “downstream” translation activities, an influential example of which is the economic assessments (Health Technology Appraisals or HTAs) of RM technologies carried out by authorities such as UK’s National Institute for Health and Care Excellence (NICE) when deciding whether to adopt the technology within a health-care system. An HTA is a means of ensuring that only cost-effective technologies will be adopted, that is, only those technologies whose clinical effectiveness justifies their cost (RM technologies may be high cost due to their complex manufacturing processes). Manufacturers must submit a range of data that can be used to calculate both the anticipated implementation and day-to-day cost of the technology if it were to be adopted, and the anticipated clinical and social benefits to the patient. In effect, various points of reference are used to forecast a future for the technology; it becomes entangled within future-oriented understanding as being cost-effective or not (or as being “unable to appraise” due to insufficient data). Given the gatekeeping role of HTA agencies, these entanglements can have a major impact on the success of an innovation (Regenerative Medicine Expert Group 2015).

A good example of this is ChondroCelect, an autologous-chondrocyte-based product developed by the Belgium company, TiGenix, for treating cartilage defects in the knee. In 2009, ChondroCelect was the first RM product to receive marketing authorization via the European Medicine Agency’s Committee for Advanced Therapies; existing clinical data, the committee declared, demonstrated that the product was clinically effective and had an acceptable safety profile. Yet ChondroCelect failed to be widely adopted within EU health-care systems, largely due to uncertainties about its cost-effectiveness. In the UK, a cost assessment conducted by NICE highlighted the limitations in existing data, particularly relating to longer term clinical benefit, and thus concluded that cost-effectiveness could not yet be demonstrated. It was not, then, recommended for routine use in the UK Health-care system. Similar responses from other national HTA bodies eventually led TiGenix to declare that the ChondroCelect was not commercially feasible, and it thus requested that marketing authorization be withdrawn. In this regard, ChondroCelect illustrates what appears to be a major challenge in the commercialization of RM.

The commercial (un)feasibility of a product does not necessarily mean development will come to a grinding halt, however: investors may use their access to financial resources to drive a product in a different direction, with the longer-term aim of securing higher prices on the market and so a major return on their investment (Roy and King 2016; Birch 2016). A good example of this is Prochymal, a mesenchymal stem cell-based, immunomodulatory product. The product was developed by Osiris and it underwent clinical trials for several indications with mostly disappointing results. Although approved in some jurisdictions for the treatment of graft-versus-host disease, the small patient population with this indication suggested it would have limited commercial value. The product was, nevertheless, purchased from Osiris by the Australian company Mesoblast at a cost of USD50 million and is now undergoing clinical trials for Crohn’s disease (Waltz 2013). A more recent (2015) additional investment of USD45 million has been made by Celgene, on the assumption that they can secure a broader license to treat a number of more common related conditions.

Both the ChondroCelect and Prochymal stories show how the commercialization of RM can take different directions with different outcomes depending on the involvement of clinical, regulatory, and financial actors.

## Discussion: Value Tensions across Networks

What we have shown in this article is that a strong sociotechnical imaginary of health and wealth in the UK has been institutionalized within the CGTC, and it is reflected in the way in which the Catapult attempts to build momentum around and steer the creation of prospective novel RM products. This involves a range of activities that we have described as entailing the disentangling and framing of prospective products so that they are endowed with specific futures that promise clinical utility, while meeting the interlinked challenges of regulatory approval and scale-up. We have also seen how the envisaging and attempted realization of this biovalue comes into tension with a number of competing narratives––other entanglements––such as debates relating to the patenting of biological material, the fostering of public good within UK research infrastructure through the UKSCB, and cost assessment analyses that can pose major problems for CGTC and private company plans relating to reimbursement in the clinical market.

The concept of sociotechnical imaginaries is useful in helping us to see how some narratives emerge in the play of these entanglements, and how some come into conflict, and so how certain futures are enabled and others not. We think that this concept can be given further utility by linking it to Callon’s (1994) discussion of networks and markets––specifically, the way in which the latter is performed and brought into being by the former. The tensions we describe above illustrate how sociotechnical networks can be aligned or misaligned to varying degrees.

Callon’s discussion of the distinction between “public” and “private” goods is important here. The discourse of health and wealth suggests a complementary alignment between practices that serve the public health of the community on the one hand, and private commercial interests of industry on the other hand. Callon stresses, however, that we should refrain from positing an a priori distinction between public and private; rather, we should see public goods and “private goods” and the distinctions between them as being performed––or enacted––by often complex networks of actors. The value tensions we describe above reflect these potentially conflicting networks. For Callon, the most important matter of concern is whether networks enable the circulation and socialization of knowledge, fostering accessibility to a wide range of users.

We have seen that one of the principal objectives of the CGTC is to help accelerate the passage of prospective products to the market. As this is undertaken, countervailing processes come into play that reveal the tensions between public and private actors and the difficulties faced by the CGTC as an intermediary agency that attempts to create bridges between the two. The various entanglements discussed above, related to patenting and the roles of the UKSCB and NICE, are points at which the closed/open character of forms of knowledge come into play––patenting as a form of closure, the bank as a medium for open access, and NICE as gatekeeper––closing or opening paths to the market according to comparative cost–benefit modeling. Of these three, the Catapult and its partners are most likely to be able to negotiate the value terrain of patenting and cost–benefit analysis since in both domains the task is to position knowledge claims such that they can be seen as being distinct from and adding value to the state of the art. Moreover, the Catapult and NICE may become more aligned. NICE has recently moved toward modes of assessment intended to facilitate innovation, such as “progressive value assessment” and “productive risk sharing,” thus reflecting a perspective that is more in line with the CGTC’s “imaginary,” at least in broad terms. The more difficult task relates to how to engage with the sociotechnical imaginary of the UKSCB, which is based on providing access to thoroughly characterized cell lines checked for quality and safety. This characterization and checking enables, rather than constrains, access to and movement of quality-assured lines in a way that maximizes the utility of lines as a public good resource. In contrast, the free exchange of cell lines among researchers (i.e., without mediation by a bank) is known to have resulted in millions of dollars and euros wasted on research due to lack of quality assurance (e.g., lines may not be properly authenticated or cross-contamination occurs) and the duplication of effort (Geraghty et al. 2014). In terms of Callon’s argument, the values enacted by the UKSCB foster not an unfettered circulation of lines but one anchored in quality assurance. How the CGTC addresses this question now and in the future is an especially important challenge to its sociotechnical imaginary, since, while acknowledging that the quality of lines is of great importance, and one that depends on researchers being able to access and test lines as third parties, the parallel weight it gives to the narrative of “wealth” recognizes the importance that firms give to exclusivity in regard to the use of the lines they have developed (Holm 2015).

## Note

1. Directive 98/44/EC of the European Parliament and of the Council of July 6, 1998, on the legal protection of biotechnological inventions.
